# Association between violence and depression during pregnancy with perinatal outcomes: a moderated mediation analysis

**DOI:** 10.1186/s12884-022-05106-y

**Published:** 2022-11-01

**Authors:** Liliana Yanet Gómez Aristizábal, Susana Cararo Confortin, Rosângela Fernandes Lucena Batista, Maria Teresa Seabra Soares de Britto e Alves, Vanda Maria Ferreira Simões, Antônio Augusto Moura da Silva

**Affiliations:** Graduate Program in Collective Health, Federal University of Maranhão, Rua Barão de Itapary, 155, MA 65020-070 São Luís, Brazil

**Keywords:** Violence, Depression, Pregnancy, Perinatal outcomes, Mediation analyses, Moderation analyses

## Abstract

**Objective:**

To assess the direct, indirect, and total effects of violence during pregnancy on perinatal outcomes, and to evaluate the effect of violence as a moderator of the mediated relationship of depression with perinatal outcomes.

**Methods:**

Data was collected from the prenatal study and follow-ups of the BRISA cohort, São Luís, Maranhão, Brazil. The perinatal outcomes investigated were: birth weight (BW), intrauterine growth restriction (IUGR) and gestational age (GA). Violence against women was evaluated using the World Health Organization Violence against Women instrument (Violence during pregnancy – regardless of the type of violence; Physical violence during pregnancy; Psychological violence during pregnancy). Depressive symptoms during pregnancy were evaluated as a mediating variable. Moderated mediation analysis was performed to estimate the effects of violence and depression on perinatal outcomes.

**Results:**

Three types of violence analyzed by depression had an indirect effect in BW and GA. None of the types of violence showed an association with IUGR. All types of violence analyzed showed a moderated mediation effect with BW and GA. Only among women who experienced violence were birth weight and gestational age lower the higher the values of depressive symptoms.

**Conclusion:**

Violence and depression are only associated with lower BW and GA when they occur simultaneously.

## Introduction

The violence experienced by women during pregnancy is considered an extremely complex phenomenon [1], and has become a major public health and human rights issue [2, 3].

Studies have shown that pregnancy represents a risk factor that accelerates episodes of violence against women and, in cases where violence already exists, the frequency and severity pattern of episodes may change [4–7].

Repercussions of violence against women during pregnancy are diverse and are associated with negative effects that affect a woman’s physical, mental, and reproductive health and social conduct, as well as the health of the fetus and newborn [8–11].

Evidence indicates that the effect of violence against women during pregnancy may be presented directly and indirectly. Directly, exposure to violence during the perinatal period increases the risk of adverse birth outcomes, such as low birth weight, preterm birth, or intrauterine growth restriction, through behavioral or physiological responses to stress, which could include the release of vasoconstrictors or cortisol, or the release of prostaglandin, which can cause premature contractions. On the other hand, women who experience violence may have little autonomy to make decisions about their health and seek care, which can lead to inadequate prenatal care and poor nutrition, which could lead to intrauterine growth restriction and low birth weight [12]. It can also have other adverse outcomes in women’s physical and mental health, such as depression, anxiety, substance abuse, among others. Indirectly, the effects can present themselves by the mediation of negative maternal situations like depression [12–15].

Depression, as one of the many consequences of violence during pregnancy, can affect women’s health, child development, and quality of life. Pregnant women who suffered from depression had high levels of stress hormones, such as cortisol, affecting fetal growth, fetal brain development, birth weight, and other newborn outcomes [16].

Violence against women and depression are a cause for concern, since evidence suggests that their joint and simultaneous occurrence during the perinatal period increases the risk of negative implications for fetal and child health, and the negative effect is expected to be greater when depression appears alongside violence during pregnancy [17].

Hence, our study assessed the direct, indirect, and total effects of violence during pregnancy on perinatal outcomes (birth weight – BW, intrauterine growth restriction – IUGR, and gestational age – GA), as well as the effect of violence as a moderator of the mediated relationship of depression with perinatal outcomes, investigating four hypotheses: H1 – Violence during pregnancy directly affects perinatal outcomes; H2 – Violence during pregnancy has a total effect on perinatal outcomes; H3 – Depression mediates the effect of violence during pregnancy on perinatal outcomes (Indirect effect); H4 – Indirect association between violence during pregnancy and perinatal outcomes mediated by depression depends on whether or not the woman experienced violence (moderated mediation effect).

## Methods

### Study design

This prospective cohort study used data from birth cohorts - BRISA (Brazilian Birth Cohort Studies) [9], registered between February 2010 and June 2011 (started in prenatal care – 22 to 25 weeks of gestation), at birth and follow-up in 2012/2013. This study used the cohort of the municipality of São Luís, Maranhão, Brazil (Da Silva, 2014).

### Study population and sampling

The study population consisted of 1,447 women in the prenatal cohort, of which 1,381 were re-interviewed within 24 h postpartum (Da Silva, 2014). Observations with no response in the exposure (violence), outcome (perinatal outcomes), and mediation (depression) variable were excluded from the sample, thus totaling 1,130 mother/newborn pairs.

### Data collection

Data was collected in two moments: prenatal care and birth, by interviews with application of structured questionnaires by properly trained personnel. The women were contacted at ultrasound and prenatal clinics and invited to participate in the study (Da Silva, 2014).

### Exposure variable

Violence against women was assessed using the World Health Organization Violence against Women instrument [18, 19], self-applied between the 22nd and 25th weeks of gestation. It consists of 26 questions, asking whether the woman has experienced violence during her current pregnancy and in the 12 months prior to pregnancy, including psychological, physical, and sexual violence.

Our analysis included violence during pregnancy (physical, psychological or sexual), physical violence during pregnancy, and psychological violence during pregnancy, assessed as “Yes” or “No.”

### Mediation variable

To identify depressive symptoms during pregnancy, we used the Center for Epidemiologic Studies Depression Scale (CES-D) [20], assessing the frequency of depressive symptoms experienced in the week prior to the interview (0 = Rarely – less than 1 day; 1 = for a short time – 1 or 2 days; 2 = during a moderate time – 3 to 4 days; 3 = during most of the time – from 5 to 7 days). For the analysis, depressive symptoms were considered as a continuous variable (final score ranging from 0 to 60 points).

### Outcome variables

**Birth weight (BW)**: birth weight (kg) was obtained from medical records.

**Gestational age** at birth **(GA)**: was considered as the complete weeks of gestation, calculated from the last normal menstrual period (LNMP) reported by the mother. Day 15 was imputed for all cases with unknown LNMP day. Cases with less than 20 and over 43 weeks were recoded as missing. Cases with missing GA were imputed in a regression model containing birth weight, parity, per capita monthly family income, and newborn sex [21, 22].

#### Intrauterine growth restriction (IUGR)

IUGR was defined by the birth weight ratio [23], which classifies as having intrauterine growth restriction newborns with values below 0.85. This ratio is calculated by dividing the newborn’s weight by the weight corresponding to the 50th percentile of the weight-for-gestational-age curve.

### Complementary variables

For adjustment, we included the following variables: mother’s age (years), number of children (continuous), maternal education (0–4, 5–8, 9–11, and 12 or more years), maternal occupation (manual labor, non-manual labor, does not work), monthly family income in minimum wage (R$ 510.00 in 2010), pregnancy planning (yes/no) and economic classification (A/B, C, D/E [24] – categories A and B having the highest spending power) [24]. To define the socioeconomic classification, the Brazilian Economic Classification Criteria, CEB, was used, which is a standard of socioeconomic classification, based on households. It consists of a way of measuring the purchasing power of the population. Thus, it is possible to segment individuals into classes. The parameter considers aspects such as the physical structure of the residence, consumer goods and education of the head of the family. The categories are classified according to the minimum wage, thus A (above 20 minimum wages), B (10 to 20 wages), C (4 to 10 wages), D (2 to 4 wages) and E (Up to 2 wages).

To describe the study sample, we included the variables: skin color/ethnicity (white, Asian, brown/mixed race, black), marital status (married, domestic partnership, single, separated, widowed), and type of delivery (cesarean, forceps, normal).

### Data analysis

The sample was characterized by means of descriptive analyses (absolute and relative frequencies, mean, and standard deviation).

**Fig. 1:**
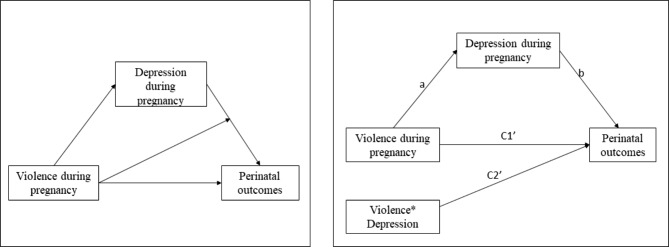
Conditional process model of violence against women and depression during pregnancy and perinatal outcomes Panel A. Conceptual Diagram. Panel B. Statistical Diagram

We also performed moderated mediation analysis (conditional process modeling), which occurs when the effect of the exposure on the outcome by a mediating variable changes depending on the levels of the moderating variable. Violence against women during pregnancy was considered as a possible moderator of the mediating relationship between depression during pregnancy and perinatal outcomes (Fig. 1).

To obtain the effects of the conditional process, we estimated three models according to type of violence (violence during pregnancy regardless of type, physical violence during pregnancy, and psychological violence during pregnancy) for each of the perinatal outcomes independently.

Bootstrapped confidence intervals (BCI) of the indirect effect were set at 95%, with a 0.05 confidence level [25]. We estimated the hypothesized conditional process model, specifying the effects of moderated mediation. The conditional process model was estimated using the R program with the interaction and mediation packages.

### Ethical aspects

This project met the criteria of the National Health Council Resolution No. 196/1996 and its complementary regulations. It was approved by the Research Ethics Committee of the University Hospital of UFMA under opinion No. 223/2009, protocol: 4771/2008-30. The interviewees were invited to participate in the research. Those who agreed signed the informed consent form (ICF).

## Results

We analyzed 1,131 mother-newborn pairs, with an average birth weight of 3,249 g and mean GA at birth of 39.3 weeks, and 15.3% of deliveries showed IUGR. Cesarean was performed in 49.9% of births, and 65.4% of pregnancies were unplanned (Table 1).

Participants’ mean age was 26.1 (± 5.4) years, 50.0% did not work, 81.5% were married or in a domestic partnership, and 76,2% had 9 to 11 years of schooling. Brown/mixed race women represented 67.5% of the study population and 68.1% belonged to economic class C (Table 1).

Overall, women had a mean depression score of 14.9 points, meaning moderate depressive symptoms. Of the women studied, 49.7% experienced violence during pregnancy, 48.5% experienced psychological violence, and 12.5% physical violence during pregnancy (Table 1).

**Table 1:** Characterization of the study sample. BRISA Birth Cohort – São Luís, Maranhão, Brazil

Variable	Category	Mean	Standard deviation
Mother’s age (years)		26.1	5.4
Number of children		1.7	0.9
Depression score		14.9	10.0
Birth weight (grams)		3.249	497.9
Gestational age (weeks)		39.3	2.0
		**n**	**(%)**
Intrauterine Growth Restriction	Yes	173	15.3
	No	958	84.7
Violence during pregnancy	Yes	562	49.7
	No	569	50.3
Physical violence during pregnancy	Yes	141	12.5
	No	990	87.5
Psychological violence during pregnancy	Yes	549	48.5
	No	582	51.5
Skin color/Ethnicity	Mixed race	764	67.6
	White	177	15.7
	Black	170	15.0
	Asian	20	1.8
Schooling	12 or more	138	12.2
	9–11 years	863	76.2
	5–8 years	102	9.0
	0–4 years	28	2.5
Marital status	Married/stable union	922	81.5
	Single/separated/widowed	209	18.5
Mother’s occupation	Manual work	375	33.2
	Non-manual work	190	16.8
	Does not work	566	50.0
Socioeconomic status	A/B	176	15.6
	C	770	68.1
	D/E	185	16.4
Pregnancy planning	No	740	65.4
	Yes	391	34.6
Type of delivery	Normal	567	50.1
	Cesarean section	564	49.9

Results of the three models evaluated proved to be largely consistent with the study hypothesis, i.e., that violence and depression during pregnancy have a negative effect on birth outcomes only when presented jointly and not separately.

**Fig. 2:**
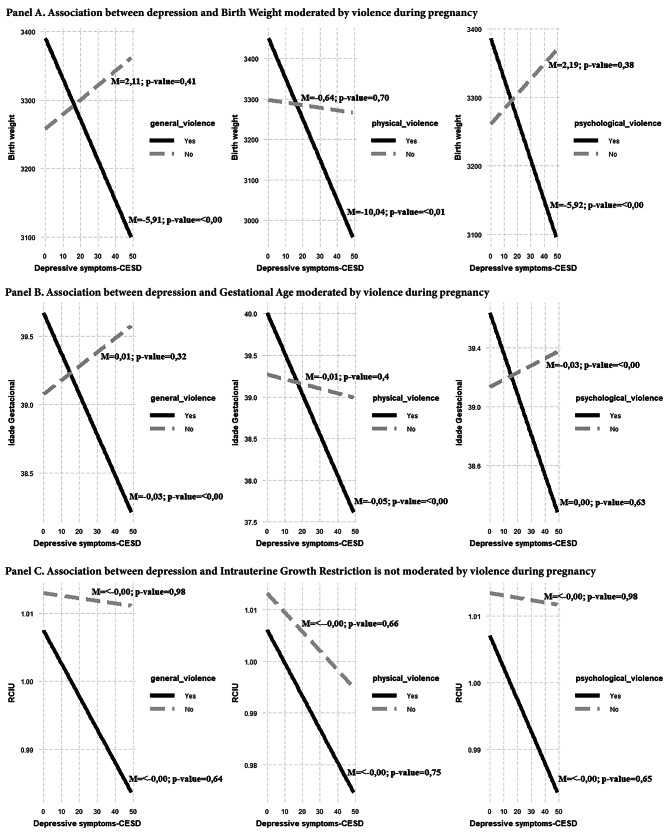
Association between depression and perinatal outcomes.

Violence showed no direct effect on birth weight (BW), gestational age (GA), and intrauterine growth restriction (IUGR). Violence showed an indirect effect on birth weight and gestational age, but not on IUGR. Birth weight and gestational age were lower the higher the values of depressive symptoms only among women who experienced violence. Depression was not associated with these perinatal outcomes if women did not experience violence (Fig. 2).

**Fig. 3:**
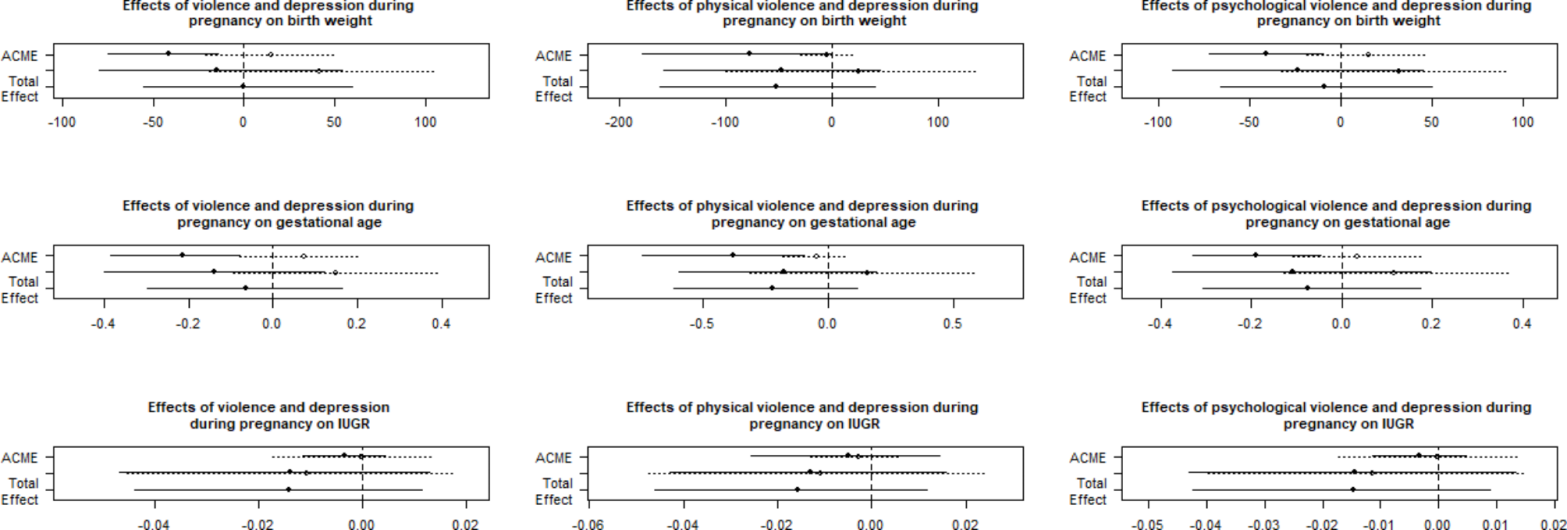
Effect for the association between violence against women and depression during pregnancy and perinatal outcomes Caption: ACME: Average causal mediation effect. ADE: Average direct effect

Regarding the indirect or mediation effect, our findings show that depression during pregnancy is a mediating variable in the relationship of the three types of violence analyzed in the treatment group (women who experienced violence) with BW and GA at birth, with physical violence having a greater effect on BW and GA when compared with the other two types (Table 2; Fig. 3).

**Table 2:** Effects for the association between violence against women and depression during pregnancy and perinatal outcomes

	Violence during pregnancy**		Physical violence**		Psychological violence**	
	**Estimate**	**p-value**	**Estimate**	**p-value**	**Estimate**	**p-value**
**Birth Weight**						
ACME (Control group)	14.85	0.41	−4.89	0.67	15.15	0.39
ACME (Exposure group)	−41.69	< 0.001	−77.72	0.05	−40.89	< 0.001
ADE (Control group)	37.59	0.19	24.16	0.67	35.66	0.30
ADE (Exposure group)	−19.95	0.71	−48.57	0.40	−20.38	0.53
Total effect	−4.10	0.98	−53.55	0.34	−5.23	0.76
MME	−56.54	0.02	−72.74	0.08	−56.04	0.02
**Gestational Age**						
ACME (Control group)	0.07	0.33	−0.04	0.51	0.03	0.67
ACME (Exposure group)	−0.21	< 0.001	−0.38	0.01	−0.19	< 0.001
ADE (Control group)	0.13	0.27	0.14	0.54	0.12	0.31
ADE (Exposure group)	−0.16	0.26	−0.19	0.35	−0.10	0.53
Total effect	−0.08	0.55	−0.24	0.20	−0.07	0.60
MME	−0.29	0.00	−0.33	0.03	−0.22	0.02
**Intrauterine growth restriction**						
ACME (Control group)	< 0.001	0.96	< 0.001	0.54	< 0.001	0.98
ACME (Exposure group)	< 0.001	0.39	< 0.001	0.63	< 0.001	0.43
ADE (Control group)	−0.11	0.46	−0.01	0.58	−0.01	0.43
ADE (Exposure group)	−0.01	0.34	−0.01	0.44	−0.15	0.31
Total effect	−0.01	0.28	−0.02	0.31	−0.01	0.26
MME	< 0.001	0.66	< 0.001	0.85	< 0.001	0.72

**Caption: ACME**: Average causal mediation effect **ADE**: Average direct effect **MME**: Moderated Mediation Effect

**Models adjusted by: Mother’s age, number of children, pregnancy planning, economic class, family income, schooling and mother’s occupation

Investigation of the direct effect of the three types of violence analyzed on BW, GA and IUGR showed no significant effect, and neither did the total effect (Table 2; Fig. 3).

Table 2 shows the results for the three models and each of the outcomes analyzed regarding the conditional indirect effect or the moderated mediation effect. For BW and GA, the three types of violence significantly moderated the mediation relationship of violence via depression. We found no moderated mediation effect of IUGR in any of the three models (Table 2; Fig. 3).

## Discussion

Our results showed an indirect effect of violence (in general) and of physical and psychological violence during pregnancy, via depression, on birth weight and gestational age at birth. We observed no direct effect of violence on these perinatal outcomes. We also found a moderated mediation effect, showing that the indirect effect of violence on birth weight and gestational age at birth, via depression, is conditional on women having experienced violence. Birth weight and gestational age were lower the higher the values of depressive symptoms only among women who experienced violence.

In our research, women who experienced violence during pregnancy were more likely to have children with lower weight and lower gestational age at birth, corroborating previous studies [26–29]. One such study showed that women who experienced domestic violence were more predisposed to preterm labor and having children with low birth weight compared to women who did not [26]. A meta-analysis found a positive association between violence and low birth weight (OR: 1.4; 95%CI: 1.1–1.8), concluding that any type of violence during pregnancy is part of a complex network of factors contributing to low birth weight [29].

We found no direct association of physical violence with perinatal outcomes, diverging from other studies [30–32]. Sigalla et al. (2017) showed that exposure to physical violence during pregnancy, after adjusting for confounding factors, quadruples the chance of low birth weight when compared with women who were not exposed to this type of violence during pregnancy [30]. Another study observed that women who experienced violence were 2.5 times more likely to have a child with low birth weight, and six times more likely to have a very low birth weight (< 1,500 g) [31].

Psychological and emotional violence showed no direct association with the higher chance of low birth weight or preterm delivery, corroborating the findings of a study that, after adjusting for confounding factors, showed that sexual and psychological violence were not associated with birth weight and preterm delivery [33].

The association between psychological violence during pregnancy and birth weight and gestational age at birth has not been agreed upon in the existing scientific literature, which can be explained by the differences in measuring this type of violence. Besides, most of the literature documents the effects of physical or sexual violence against women, disregarding psychological violence.

Our results showed an indirect or mediating effect of depression on birth weight and gestational age at birth. Different studies that have analyzed the relationship between violence and different perinatal outcomes report that this effect can be mediated by other factors, such as depression [29, 34, 35]. However, we found no studies analyzing the effect of depression as a mediating factor in the relationship of violence during pregnancy with birth weight and gestational age as in our study, limiting the comparability of the results.

We found a moderated mediation effect in all models analyzed with birth weight and gestational age at birth. Although we found no research exploring the moderated mediation effect, some studies [36–38] that jointly analyzed the effect of violence and depression during pregnancy on birth weight and gestational age at birth obtained results similar to those presented here.

Depression appears to be a major mental health response in women who experience violence, seriously impairing normal life functioning. The mediating mechanism of depression can be explained by the depressive symptoms resulting from victimization that may accentuate chronic illnesses, and cause isolation and inadequate access to health care, increased behavioral risks (like smoking and alcohol abuse), or inadequate maternal nutrition. Moreover, activation of hypothalamic-pituitary-adrenal or placenta-adrenal neuroendocrine axes related to the depressive state may affect uteroplacental blood flow, which relates to gestational age and birth weight [29, 39].

Women with unfavorable fetal outcomes were more predisposed to suffer depression and to have experienced partner violence during pregnancy. Women with children born with low birth weight were especially more likely to have experienced violence and depression when compared with mothers of children with normal birth weight [37]. Another exploratory study assessed the relationship between intimate partner violence, depression and post-traumatic stress as additional predictors of birth weight, showing that partner violence was more associated with low birth weight among women who also suffered from depression or post-traumatic stress disorder [40].

As for limitations, the present study did not analyze partner violence during pregnancy, while most studies analyze this type of perpetrator of violence against women, limiting this comparability. Our study also did not evaluate sexual violence, as only a small number of women reported it.

Divergent results from the studies may be due to sample size, the inclusion or not of adjustment factors, different methods of measuring violence and depression, and exposures grouped into different categories. Regarding violence, the studies clearly diverge because they are limited to analyzing physical and sexual violence, not including psychological violence, to estimate the effects on perinatal outcomes. Other contextual and individual factors that determine women’s perception of the definition of violence and depression can also play a role, being conditioned to each woman’s experiences and culture.

Another strong point to consider is the use of a moderated mediation model. Contemporary research issues in the social sciences increasingly involve complex relationships between multiple variables that operate together and that, in some cases, arise when their associations are conditional on other variables, as in our study, which include violence and depression during pregnancy. Integrating mediation and moderation into a single model allows to examine even more differentiated relationships and establish conditional effects.

To our knowledge, this study is one of the few cohort studies that has evaluated the effect of moderated mediation between violence and depression during pregnancy on weight and gestational age at birth, by establishing direct, indirect, total, and conditional indirect or moderated mediation effects.

Experiencing violence and mental health issues, especially depression, during pregnancy has been associated with threats to the child’s health. Despite being phenomena that often appear together, most studies have analyzed the specific effect of these exposures separately, ignoring the moderating effect they may have on birth outcomes.

In conclusion, we found that when violence and depression coexist, lower BW and GA may occur.

## Data Availability

The data that support the findings of this study are available from e-mail rosangela.flb@ufma.br, but restrictions apply to the availability of these data, which were used under license for the current study, and so are not publicly available. Data are however available from the authors upon reasonable request and with permission of Rosangela Fernandes Lucena Batista.

## Declarations

### Authors’ information

**LYGA**: PhD, postdoctoral fellow at the Postgraduate Program in Public Health at the Federal University of Maranhão (UFMA). Has experience in Public Health, with emphasis on Epidemiology, working mainly on the following topics: people with disabilities, knowledge management, information and health of women and children, breastfeeding, violence against women and depression during and after pregnancy, sexual and reproductive health of migrant women. **SCC**: PhD, postdoctoral fellow at the Postgraduate Program in Public Health at the Federal University of Maranhão. Member of the Epifloripa research group and the Public Health Research Group at the Federal University of Maranhão. She works in research in Physical Education and Public Health, with an emphasis on Epidemiology and in the areas of Life Cycle, Sarcopenia and Health-related Physical Activity. **RFLB**: PhD, professor at the Department of Public Health and permanent professor at the Graduate Programs in Public Health and Nursing at the Federal University of Maranhão. She has experience in Public Health, with emphasis on Epidemiology, working mainly on the following topics: sleep, child growth, child mortality, prematurity, cesarean section and violence against women. She coordinates the fieldwork of the cohort studies in São Luís (BRISA and RPS study). **MTSSBA**: PhD, Professor at the Federal University of Maranhão. She has experience in the field of Medicine, with an emphasis on Public Health, working mainly on the following topics: Evaluation of Health Services, HIV, HIV / AIDS, Assistance to women, Quality of care and Childbirth assistance. **VMFS**: PhD, professor at the Department of Public Health and the Postgraduate Program in Public Health at the Federal University of Maranhão. Has experience in Pediatrics, Collective Health and Maternal and Child Health, working mainly on the following topics: growth and development, epidemiological studies, cohort studies and chronic non-communicable diseases, child and adolescent health, newborns at risk, preterm birth, low birth weight, infant mortality. **AAMS**: PhD, professor at the Department of Public Health and the Postgraduate Program in Public Health at the Federal University of Maranhão. Has experience in Public Health, with emphasis on Epidemiology, working mainly on the following topics: low birth weight, infant mortality, prematurity, cesarean section and obesity. Participates in 5 birth cohort studies in Brazil and a cohort study of children with Congenital Zika Syndrome.
